# Immune checkpoints inhibitors in the treatment of progressive multifocal leukoencephalopathy: A systematic review and meta-analysis

**DOI:** 10.1007/s10072-026-09498-z

**Published:** 2026-11-04

**Authors:** Ahmad Toubasi, Thuraya N. Al-Sayegh

**Affiliations:** 1 Department of Neurology, Vanderbilt University Medical Center (VUMC), 1211 Medical Center Drive, Nashville, TN 37232 USA; 2 Department of Internal Medicine, University of California San Francesco, Fresno, CA USA

**Keywords:** Progressive multifocal leukoencephalopathy, Multiple sclerosis, Cancer, Immune checkpoint inhibitors

## Abstract

**Background:**

Progressive multifocal leukoencephalopathy (PML) is a rare but catastrophic demyelinating disease of the central nervous system (CNS) caused the JC virus (JCV). However, no effective treatments have been identified. Immune check point inhibitors (ICIs) are considered a potential treatment option. We conducted a systematic review and meta-analysis to pool the data from the literature that evaluates the efficacy of using ICI in the treatment of PML.

**Methods:**

The search was conducted on January 24, 2026 using PubMed, Scopus, Cochrane Central Register of Controlled Trials (CENTRAL), and Embase. Studies were included if they included patients with PML and evaluated the efficacy of using ICIs including PD-1, PD-L1, and CTLA-4 inhibitors in PML treatment. The exposure of interest was the use of ICI in the treatment of PML. The outcomes of interest were survival, disability and cerebrospinal fluid (CSF) viral load. Rate and weighted mean difference (WMD) and their 95% confidence intervals (95%CI) were used as the effect size in the analysis.

**Results:**

We included a total of 186 patients with PML from 52 articles. The survival rate among the included patients was 62% (95%CI: 52%-72%). There was no significant difference in disability between before and after the treatment with ICI (WMD =− 0.03; 95%CI: − 2.11–2.04). On the other hand, the treatment was associated with lower viral levels in the CSF (WMD = 69.20; 95%CI: 1.69–136.70).

**Conclusions:**

Future randomized clinical trials or comparative observational studies that investigate the efficacy of using ICI in the treatment of PML compared to other treatment modalities are needed.

**Supplementary Information:**

The online version contains supplementary material available at https://doi.org/10.1007/s10072-026-09498-z.

## Introduction

Progressive multifocal leukoencephalopathy (PML) is a rare but disastrous demyelinating disease of the central nervous system (CNS). The disease is caused by reactivation of JC polyomavirus (JCV) in immunocompromised patients [1]. JCV is usually asymptomatic, lifelong persistent or latent infection in the general population that mostly occurs in childhood [2]. The virus establish low-level persistent or latent infection in the kidney and bone marrow derived cells [1, 3]. However, once the immune system is suppressed, JCV reactivates and replicate vigorously in oligodendrocytes to cause lytic infection of the CNS glial cells resulting in PML [1]. The acquired immunodeficiency syndrome (AIDS) pandemic led to the first surge in the occurrence of PML [1]. The implementation of antiretroviral therapies decreased the incidence of PML [1]. With the development of modern treatments for cancers and multiple sclerosis (MS), organ transplantation and chronic inflammatory diseases, the incidence of PML increased again [1]. Several studies investigated the prevalence of the underlying conditions among patients with PML and reported that 44%-49% of patients were diagnosed with human immunodeficiency virus (HIV) infection, 22%-31% were diagnosed with hematologic malignancies, 9%-20% with chronic inflammatory diseases and 3–4% with solid organ transplant [4–6]. The mortality rate associated with the disease is very high ranging from 75% among patients with HIV [7] and 90% among patients without HIV infection [8]. Even among survivors, the majority (70%) suffer from substantial disability which is moderate to severe in 25%-50% of them [9] with up to 44% of the survivors developing seizure disorder [10].

Given the high mortality associated with PML and the absence of effective antiviral therapies, several therapeutic strategies have been explored, including the development and repurposing of existing medications. Examples include interleukin‐7 (IL‐7) [11], adoptive transfer of anti‐polyomavirus specific T cells [12], maraviroc [13], and immune checkpoint inhibitors (ICIs) [14]. ICIs are considered a promising medication in the treatment of PML. Inhibitory immune checkpoint molecules such as programmed cell death‐1 (PD‐1), PD ligand-1 (PD-L1) and cytotoxic T-lymphocyte-associated protein 4 (CTLA-4) are expressed by activated T cells and play a vital role in the maintenance of peripheral tolerance [15]. ICIs inhibit these molecules mounting an immune response against diseases such as cancer and chronic infections.

Treatment options for PML remain limited, and given its devastating short- and long-term outcomes, further advances in therapeutic strategies are urgently needed. However, it is still unclear whether ICI are effective in the treatment. This is mainly due to the fact that most of the published literature consists of small case reports and case series and no systematic review or meta-analysis have been done to pool this data and produce stronger level of evidence.

To address these aims, we conducted a systematic review and meta-analysis to pool the data from the literature that evaluate the efficacy of using ICI in the treatment of PML. We hypothesize that ICI would be associated with higher survival prevalence, and reduction in disability and JCV titers.

## Methods

### Registration and protocol

The study was conducted in concordance with the preferred reporting items for systematic reviews and meta-analysis (PRISMA). The study was prospectively registered in the international prospective register of systematic reviews (PROSPERO#CRD420261308634).

### Search strategy

The search was conducted on January 24, 2026 using PubMed, Scopus, Cochrane Central Register of Controlled Trials (CENTRAL), and Embase. The following keywords immune check point inhibitors and PML along with their related MeSH terms were used. The full search strategy is described in the supplementary material. The search was conducted by AAT. The search results were uploaded on Rayyan [16] a tool for screening articles for systematic reviews and meta-analyses.

### Study selection

The inclusion criteria included (1) case reports, case series, cohort, case–control, and clinical trials that (2) included patients with PML and (3) evaluated the efficacy of using ICIs, including PD-1, PD-L1, and CTLA-4 inhibitors, in PML treatment.

Studies were excluded if they were reviews or editorials, or if they evaluated modalities of treatment of PML other than ICIs.

Duplicates detection was done by Rayyan then checked manually by AAT. The study selection was done on Rayyan by AAT and TNA independently and any disagreements were solved by discussion [16].

### Exposures and outcomes

The exposure of interest was the use of ICI in the treatment of PML. The outcomes of interest were survival, disability and cerebrospinal fluid (CSF) viral load. Survival was defined based on the patient being alive at the last follow-up performed by the study. The disability outcome was defined as the change in disability scored using the modified rankin scale at the last follow up after treatment compared to baseline. CSF viral load was defined as the change in the JCV DNA levels in the CSF at the last follow up after treatment compared to baseline. No restrictions on the follow up time were made because it was expected that there would be variability in the follow up time among the included studies.

### Data extraction

The data of interest were extracted from the included papers using a predetermined sheet. The variables of interest included authors information, study design, sample size, the used ICI course, condition predisposing to PML, the number of patients who survived at last follow up, the mean disability scores at baseline and last follow up, and the mean JCV DNA levels at baseline and last follow up.

### Quality assessment

The quality assessment of the included papers were assessed based on their design. We used the New-castle Ottawa scale (NOS) for cohort, case–control, and cross-sectional studies while the Joanna Briggs Institute (JBI) checklist was used for case reports and case series. NOS is a scale that consists of three components including the selection of the included population, comparability and the assessment of outcomes. The score ranges between 0 and 9 with scores higher than 5 indicates good quality study. The methodological quality of case reports and case series was assessed using the Joanna Briggs Institute (JBI) critical appraisal checklists. The case-report and case-series checklists comprise 8 and 10 items, respectively. Each item was assessed as ‘Yes,’ ‘No,’ ‘Unclear,’ or ‘Not applicable.’ For the purposes of this review, studies meeting ≥ 80% of applicable criteria were considered to have high methodological quality.

The quality assessment of the included studies was done by AAT and TNA independently and any disagreements were solved through discussion.

### Data analysis

We used rate and weighted mean difference (WMD) and their related 95% confidence intervals (95%CI) as the effect measures in the analysis [17]. First, the survival rate was assessed using the rate and its 95%CI. The rate models were done using the random effect model with double arcsine transformation. Second, the WMD and its 95%CI were used to assess the changes in disability and CSF JCV DNA levels. The WMD models were also pooled using the random effect model. The heterogeneity of the models was assessed using Cochrane Q, I^2^ statistics and τ^2^. MetaXL (EpiGear Int Pty Ltd.) version 5.3 software was used in the analysis.

## Results

### Search results

**Fig. 1:**
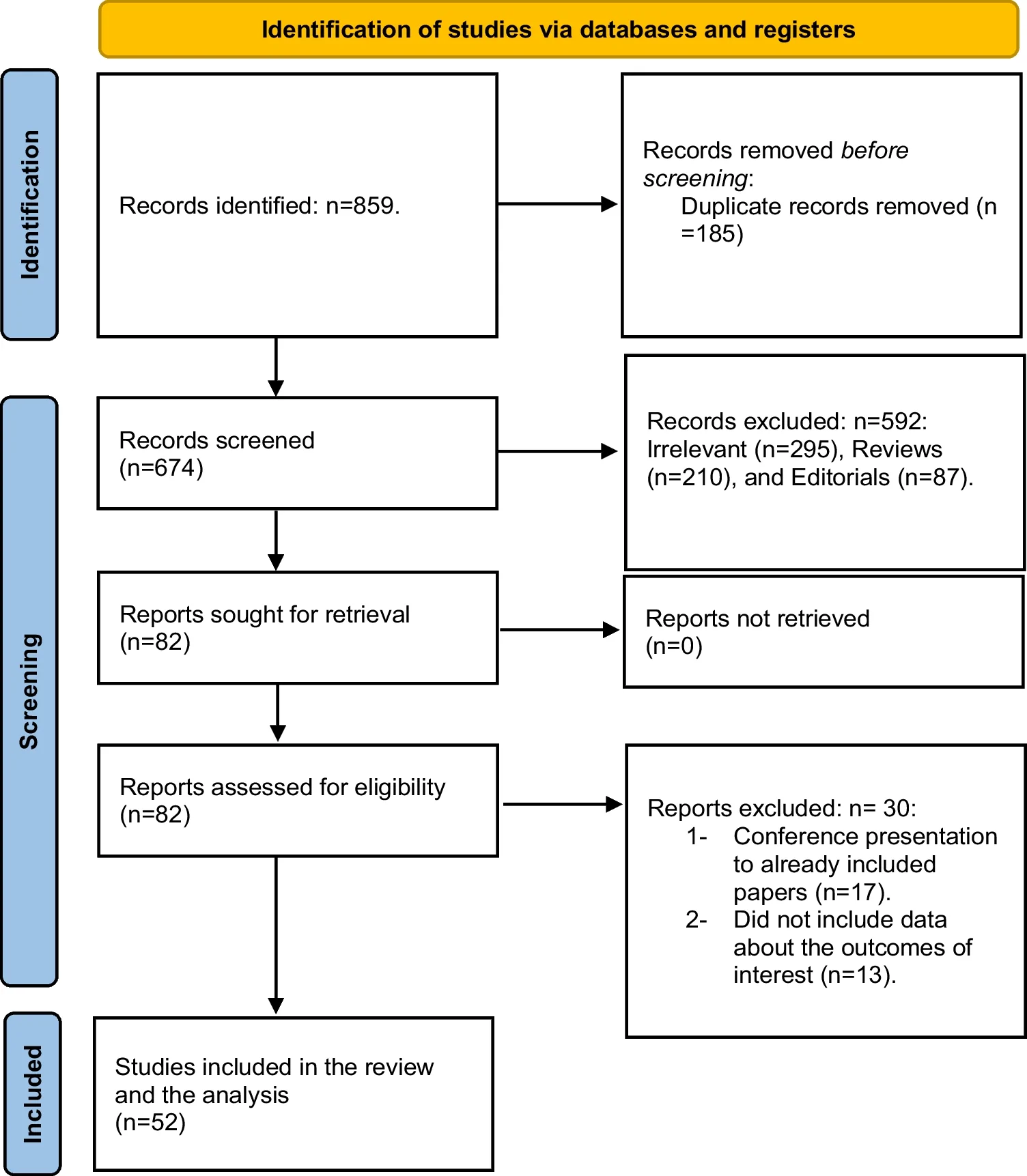
The preferred reporting items for systematic reviews and meta-analysis (PRISMA) flow chart. The figure demonstrates the selection process of the studies along with the reason of their exclusion in each step

The search yielded 859 articles. Of which, 185 papers were duplicates. The remaining 674 articles were screened using their titles and abstracts and 592 articles were excluded because they were irrelevant, reviews, or editorials. The remaining 82 articles were evaluated in their full-text form and 30 articles were excluded because they did not include data about the outcomes of interest or were abstracts for already included papers. Finally, 52 articles were included in the final analysis. Figure 1 demonstrates the study selection process.

### Characteristics of the included studies

We included a total of 186 patients with PML from 52 articles. The majority of the articles were case reports (*n* = 42), followed by case series (*n* = 8) and retrospective cohort (*n* = 2). The mean age of the included patients was 57.7 ± 12.97. The quality assessment of the included studies showed that 13.5% were of good quality (NOS > 5 or JBI ≥ 80% of applicable criteria). The majority of the papers lost points due to lack of reporting data on disability of the patients or CSF JCV viral load. The characteristics and quality assessment of the included articles are described in Table 1.

**Table 1:** The characteristics of the included studies

Last author., year	Design	Sample size	Male: Female	Mean age	Condition led to PML	Treatment course	Follow up duration	Quality assessment
Lambert et al. 2022	Case report	1	Female	77	B-cell chronic lymphocytic leukemia treated with chlorambucil and obinutuzumab	Atezolizumab 1200 mg every 3 weeks	NA	7
Kapadia et al. 2020	Case report	1	Female	69	DLBCL on RCHOP	Pembrolizumab for 4 infusions	4	5
Chiuchiarelli et al. 2025	Case series	4	3	-	HIV	Pembrolizumab 2 mg/kg every month	NA	5
Johnson et al. 2025	Case report	1	Female	75	SLE	Pembrolizumab	18	6
Wallace et al. 2024	Case report	1	Female	36	SLE on Belimumab	Pembrolizumab	NA	5
Ozturk et al. 2024	Case report	1	Male	27	Sarcoidosis	Pembrolizumab 2 mg/kg for 8 infusions	36	7
Ercan et al. 2024	Case report	1	Male	34	RRMS treated with natalizumab	Pembrolizumab 200 mg/day for 5 infusions	3	6
Jeantin et al. 2024	Case series	2	Two males	59	Liver cirrhosis	Pembrolizumab 2 mg/kg for 3 infusions and Pembrolizumab 2 mg/kg for 7 infusions	12	5
Rothenberg et al. 2024	Case report	1	Female	65	None	Pembrolizumab 200 mg every 3 weeks	10	4
Hamlyn et al.	Case series	11	1	42	HIV	Pembrolizumab	NA	6
Jeantin et al. 2024	Case report	1	Female	61	RRMS treated with fingolimod	Pembrolizumab 2 mg/kg for 3 infusions	24	6
Varmpompiti et al. 2023	Case report	1	Male	67	None	Pembrolizumab 2 mg/kg for 3 infusions	3	4
Fan et al. 2023	Retrospective cohort	4	All males	38.8	CID	Pembrolizumab 200 mg for 3–4 infusions	NA	9
Sheng et al. 2023	Case report	1	Male	77	STAT3 mutation	Pembrolizumab 2 mg/kg	NA	3
Boumaza et al. 2022	Retrospective cohort	91	1.93	62	Malignancy, HIV, and Transplant recipients	Pembrolizumab, Nivolumab, and atezolizumab 3 mg/kg	NA	5
Barritt et al. 2022	Case report	1	male	62	RRMS on Fingolimod	Pembrolizumab 200 mg for 3 infusions	36	7
Hanna et al. 2022	Case report	3	2	60	Bullous pemphigoid on rituximab and DLBCL on RCHOP, mantle cell lymphoma on rituximab	Pembrolizumab 200 mg for 3 infusions	NA	4
Varmpompiti et al. 2022	Case report	1	Male	67	HIV	Pembrolizumab 1 mg/kg for 3 infusions	6	4
Robinson et al. 2022	Case report	1	Female	52	RRMS on natalizumab	Pembrolizumab	NA	3
Lambert et al. 2022	Case report	1	Male	66	None	Atezolizumab 1200 mg	9	7
Pinnetti et al. 2022	Case series	5	4	41	HIV		NA	9
Beatric et al. 2022	Case report	1	Male	43	HIV	Pembrolizumab 2 mg/kg for 2 infusions	NA	4
Volk et al. 2022	Case series	5	Male	48	CID, DLBCL, CD40-ligand deficiency	Pembrolizumab 2 mg/kg for 2 infusions	NA	4
Darcy et al. 2021	Case report	2	Male	66	RA on rituximab and CLL on obinutuzumab	Pembrolizumab 2 mg/kg for 3 infusions	NA	4
Locati et al. 2021	Case report	1	Male	62	Head and neck cancer	Nivolumab 240 mg for 10 infusions	NA	9
Chatterjee et al. 2022	Case report	1	Male	64	NSCLC and HIV	Pembrolizumab 2 mg/kg	NA	4
Mackenzie et al. 2021	Case report	1	Male	41	DLBCL	Pembrolizumab 200 mg for 3 infusions	18	6
Lan et al. 2021	Case report	1	Male	62	SLE on rituximab	Pembrolizumab 2 mg/kg for 3 infusions	12	7
Coltart et al. 2021	Case report	1	Female	34	HIV	Pembrolizumab 2 mg/kg for 4 infusions	NA	4
Segala et al. 2021	Case report	1	Male	71	HIV	Pembrolizumab 2 mg/kg for 8 infusions	NA	4
Fischbach et al. 2021	Case report	1	Male	65	HIV	Nivolumab 240 mg for 20 infusions	NA	6
Beudel et al. 2021	Case report	1	1	65	HIV	Pembrolizumab 200 mg for 3 infusions	NA	4
Roos-weil et al. 2021	Case report	6	2	61	HIV, CVID, MG, DLBCL, Follicular lymphoma, and AML	Nivolumab or Pembrolizumab 1 mg/kg-4 mg/kg for 1–5 infusions	NA	6
Haroche et al. 2020	Case report	1	Male	70	Erdheim-Chester disease	Nivolumab 240 mg for 2 infusions	NA	4
Patel et al. 2021	Case report	1	Male	78	PPMS on ocrelizumab	Pembrolizumab 200 mg	NA	4
Stogbauer et al. 2021	Case report	1	Female	54	DLBCL on RCHOP	Pembrolizumab 2 mg/kg for 3 infusions	NA	6
Togher et al. 2021	Case report	1	Female	53	Pulmonary sarcoidosis	Pembrolizumab 2 mg/kg for 3 infusions	NA	7
Mohn et al. 2021	Case series	3	2	74	Mantle cell lymphoma, immunocytoma and DLBCL	Pembrolizumab 2 mg/kg for 3 infusions	NA	9
Holmes et al. 2020	Case report	1	Female	61	DLBCL on RCHOP	Pembrolizumab 2 mg/kg for 3 infusions	NA	6
Dufour et al. 2020	Case report	1	Male	67	Follicular lymphoma on Rituximab	Pembrolizumab 2 mg/kg for 4 infusions	NA	5
Ruiz et al. 2020	Case report	1	Female	44	HIV	Pembrolizumab 2 mg/kg for 3 infusions	NA	5
Grassi et al. 2020	Case report	1	Male	69	Sezary syndrome	Nivolumab 240 mg for 3 infusions	NA	5
Goereci et al. 2020	Case report	1	Male	71	None	Pembrolizumab 2 mg/kg for 3 infusions	NA	7
Chu et al. 2020	Case report	1	Male	52	GVHD	Pembrolizumab 2 mg/kg for 3 infusions	NA	5
Pawlitzki et al. 2019	Case report	1	Male	38	CID	Pembrolizumab 2 mg/kg for 2 infusions	NA	5
Kupper et al. 2019	Case report	1	Male	42	CID	Pembrolizumab 2 mg/kg for 2 infusions	NA	5
Walter et al. 2019	Case report	1	Female	60	None	Nivolumab 240 mg	NA	7
Rauer et al. 2019	Case report	1	Female	-	DLBCL on Rituximab	Pembrolizumab 2 mg/kg for 5 infusions	NA	7
Cortese et al. 2019	Case series	8	2	60	CLL, non-Hodgkin lymphoma, HIV, and idiopathic lymphopenia	Pembrolizumab 2 mg/kg for 3 infusions	NA	9
Hoang et al. 2019	Case report	1	Female	65	DLBCL on RCHOP	Nivolumab 3 mg/kg	NA	4
Medrano et al. 2019	Case series	3	-	-	Kidney transplant	Nivolumab 3 mg/kg for 3 infusions	NA	6
Orum et al. 2024	Case report	1	Female	50	SLE	Pembrolizumab 2 mg/kg for 2 infusions	NA	4

*AML* Acute myeloid leukemia; *CID* Combined immunodeficiency; *CLL* Chronic lymphocytic lymphoma; *DLBCL* Diffuse large B-cell lymphoma; *GVID* Graft versus host disease; *HIV* Human immunodeficiency virus; *kg* Kilogram; *MG* Myasthenia gravis; *mg* Milligram; *NSCLC* Non-small cell lung cancer; *PPMS* Primary progressive multiple sclerosis; *RA* Rheumatoid arthritis; *RCHOP* Rituximab, cyclophosphamide, doxorubicin, and vincristine; *RRMS* Relapsing remitting multiple sclerosis; *SLE* Systemic lupus erythematosus

### Survival rate among patients with progressive multifocal leukoencephalopathy

The model that evaluated the survival rate among the included patients included 52 articles and demonstrated a survival rate of 62% (95%CI: 52%-72%); the heterogeneity of the model was moderate but significant (I^2^ = 29%, τ^2^ = 0.123, *p* = 0.03).

Subgroup analysis among males included 26 articles and demonstrated a survival rate of 55% (95%CI: 41%-70%); the heterogeneity of the model was not significant (I^2^ = 1%, τ^2^ = 0.007, *p* = 0.44). Subgroup analysis among females included 16 articles and demonstrated a survival rate of 70% (95%CI: 50%-87%); the model had low and insignificant heterogeneity (I^2^ = 0%, τ^2^ = 0.000, p = 0.75).

Subgroup analysis among patients with malignancy included 11 articles and found a survival rate of 61% (95%CI: 36%-84%); the model had low and insignificant heterogeneity (I^2^ = 0%, τ^2^ = 0.000, *p* = 0.49). Analysis among patients with immunodeficiencies included 16 articles and found a survival rate of 63% (95%CI: 48%-76%); the heterogeneity of the model was low and insignificant (I^2^ = 0%, τ^2^ = 0.000, *p* = 0.83). Subgroup analysis among patients with autoimmune diseases included 6 articles and demonstrated a survival rate of 64% (95%CI: 29%-93%); the heterogeneity of the model was low and insignificant (I^2^ = 0%, τ^2^ = 0.000, *p* = 0.42). Subgroup analysis among patients with MS included 5 articles and found a survival rate of 76% (95%CI: 36%-100%); the heterogeneity of the model was low and insignificant (I^2^ = 0%, τ^2^ = 0.000, *p* = 0.56).

### Treatment effect on disability among patients with progressive multifocal leukoencephalopathy

The model that evaluated the change in disability included 8 articles. The model did not find a significant difference in disability between before and after the treatment with ICI (WMD =− 0.03; 95%CI: − 2.11–2.04); the model had low and insignificant heterogeneity (I^2^ = 0%, τ^2^ = 0.000, *p* = 1.00). Table 2 demonstrates the characteristics of the studies that evaluated the change in disability.

**Table 2:** Characteristics of the studies that evaluated the impact of ICI on patients disability

Last author., year	Sample size	Pre-treatment disability ICI measured by mRS	Post-treatment disability ICI measured by mRS
Ozturk et al. 2024	1	2	1
Fan et al. 2023	4	4.75	3.5
Boumaza et al. 2022	79	3	3
Locati et al. 2021	1	3	2
Mackenzie et al. 2021	1	22	29
Roos-weil et al. 2021	6	3.8	4.6
Mohn et al. 2021	3	2.5	2
Cortese et al. 2019	8	3.25	3.25

*ICI* Immune checkpoint inhibitors; *mRS* Modified rankin scale

### Treatment effect on viral load among patients with progressive multifocal leukoencephalopathy

The model that evaluated the change in the viral load included 22 articles and found significantly lower viral levels in the CSF after treatment compared to before it (WMD = 69.20; 95%CI: 1.69–136.70); the model had high and significant heterogeneity (I^2^ = 66%, τ^2^ = 4.670, *p* = 0.00).

Subgroup analysis among males included 11 articles and found no significant changes in the viral levels of the CSF (WMD = 149.45; 95%CI: − 28.66–327.56); the model had moderate and significant heterogeneity (I^2^ = 45%, τ^2^ = 18.838, *p* = 0.05). Subgroup analysis among females included 7 articles and the model showed no significant changes in the viral levels of the CSF (WMD = 37.34; 95%CI: − 20.64–95.32); the model had moderate and insignificant heterogeneity (I^2^ = 38%, τ^2^ = 1.150, *p* = 0.14).

Subgroup analysis among patients with malignancy included 5 articles and found no significant changes in the viral levels of the CSF (WMD = 15.12*10^3^; 95%CI: − 26.72*10^3^–32.91*10^3^); the model had high but insignificant heterogeneity (I^2^ = 55%, τ^2^ = 160.462, *p* = 0.06). Subgroup analysis among patients with immunodeficiency also included 5 articles and found no significant changes in the viral levels of the CSF (WMD = 260.61; 95%CI: − 319.10–840.32); the model had high and significant heterogeneity (I^2^ = 74%, τ^2^ = 205.921, *p* = 0.00). Subgroup analysis among patients with autoimmune diseases included 2 articles and found no significant changes in the viral levels of the CSF (WMD = 267.50; 95%CI: − 595.04–1130.04); the model had moderate but insignificant heterogeneity (I^2^ = 48%, τ^2^ = 254.752, *p* = 0.17). Subgroup analysis among patients with MS included 3 articles and found no significant changes in the viral levels of the CSF (WMD = 2.00*10^3^; 95%CI: − 3.89*10^3^–7.89*10^3^); the model had moderate and insignificant heterogeneity (I^2^ = 49%, τ^2^ = 134.880, *p* = 0.14).

## Discussion

The use of ICI has revolutionized the treatment of several cancers and improved the patients’ survival. In this systematic review and meta-analysis, we evaluated the effect of using ICIs among patients with PML. Our analysis demonstrated several findings. First, we found a survival rate of 62% in our analysis with rate of survival being variable depending on the underlaying disease. Second, there was no change in the disability between before and after treatment. Third, treatment with ICI was associated with lower JCV viral load in the CSF.

The overall survival rate among the included patients was 62%. A previous study that included patients with PML from South Korea between 1999 and 2021 found an overall survival of 57.4% [18]. A retrospective cohort among HIV patients in China found a survival rate of 78%. Another study conducted in Spain among patients with HIV included 118 patients and found a survival rate of 64% [19]. Overall, the survival rate from our cohort is similar to the survival rates in previous cohorts treated with different medications.

In our analysis, the survival rate among females was 70% compared to 55% among males. A previous retrospective paper that recruited patients with PML from South Korea between 1999 and 2021 found an overall survival rate of 57.4% without difference in the survival rate between males and females [18]. Similarly, a retrospective study that included a total of 71 patients with HIV in China found a survival rate of 78% without a difference between males and females [19]. Our analysis add to the existing literature by demonstrating that females might have better prognosis of the disease compared to males. From a pathophysiological standpoint, this could be due to the impact of sex hormones on immune regulation. Generally, females are more able to mount humoral and cellular T-cell response [20, 21].

Based on the disease predisposing to PML, the survival rate was 76% among patients with MS, 64% among patients with autoimmune diseases, 63% among patients with immunodeficiencies and 61% among patients with malignancy. In a previous study that included 68 patients with PML between 1999 and 2021 demonstrated that the survival rate was as low as 20% especially among the patients without HIV [22]. In another similar analysis among patients without HIV, the survival rate was 40% [23]. In contrast, a multi-center study in Spain that included patients with HIV who developed PML demonstrated a survival rate of 63.6%. Moreover, a study that analyzed data from the first 35 postmarketing cases of natalizumab associated PML found a survival rate of 71% [24]. Overall our data and those of others confirm that the survival rate is highest among patients with MS and autoimmune diseases compared to patients with immunodeficiencies and malignancies. These findings can be explained by the severity of the predisposing disease and the pathophysiology of ICI in managing PML. Patients with malignancies suffer from immunosuppression due to cancer related treatments which limit the immune system ability in fighting the infections by limiting the synthetic ability of the bone marrow [25]. Similarly, patients with HIV or immunodeficiencies suffer from functional or numerical depletion of immune cells resulting in the lack of ability in fighting infections [26]. In contrast, patients with autoimmune diseases and MS have dysregulated and over-stimulated immune system that is being suppressed through immunosuppressants that usually acts peripherally rather than centrally which help the immune system to bounce back faster and stronger to fight the infection after stopping the immunosuppressants and starting ICIs [27].

Our analysis demonstrated a reduction in JCV viral load in the CSF after treatment with ICI compared to before it. Checkpoint molecules such as PD-1, PD-L1 and CTLA-4 play a vital role in the maintenance of peripheral tolerance [15]. Experimental evidence suggests that the upregulation of PD-1/PD-L1 and CTLA-4 by nonhematopoietic cell types, during acute viral infection protects surrounding tissues from excessive primary T‐cell responses [28]. However, in the setting of chronic antigen stimulation such as chronic infection, the upregulation of PD-1/PD-L1 and CTLA-4 to protect healthy tissues trigger an inhibitory signaling pathway that result in the T-cell dysfunction and exhaustion, resulting in viral immune evasion [29]. However, it is important to highlight that differences in the measurement timing and assay differences. It is also vital to highlight that while we found reduction in viral load, we did not find reduction in disability which might indicate that the viral load reduction was not associated with clinical improvement.

Our analysis did not find a significant change in disability after treatment with ICI despite reduction in mRS score mean by 0.03 with a confidence interval of decrease in mRS by 2 or increase by 2. While there is no data to challenge our findings with, we hypothesize that the lack of significant change in disability might be due to several factors. First, the short follow up in the included studies compared to the time the CNS need to recover from an injury which takes months to years [30]. Second, it is possible that the study is under powered to detect difference in disability. Third, it is also possible that the injury caused by PML involves axonal degeneration and loss which our brains have more limited ability to repair if any at all [31]. Thus, initiation of treatment at advanced disease stage, might have limited benefit. However, it is also important to consider the side effects of ICI including neurological immune-related adverse events. A previous systematic review and meta-analysis that included 8,826 patients with cancer from 31 clinical trials demonstrated an incidence of 1.78 × 10–3% [32].

Before concluding, few limitations should be acknowledged. First, the lack of comparative evidence limits the strength of our findings and limits our ability to understand whether ICI impacts survival rate or not. Second, the low sample size is another limitation. Moreover, we were not able to investigate confounding factors to our analysis such as sex as studies did not provide data among males compared to females. Another limitation is that studies did not report data from time to event regarding survival, thus we were not able to conduct analysis using hazard ratio. Additionally, the survival rate analysis is limited by pooling rates at different times based on the study final follow up time and we were not able to use a specific data point because individual data from the studies were not available. Multi-center registries of PML are needed to better understand the differential survival rate based on the treatment as well as other factors.

In conclusion, our analysis demonstrated a survival rate of 62% among patients with PML treated with ICI. In addition, ICIs were associated with reduction in JCV CSF viral load. It is important to interpret our findings with caution as the survival rate does not indicate treatment efficacy. Also, the reduction in the JCV CSF viral load is of uncertain clinical significance. Future randomized clinical trials or comparative observational studies that investigate the efficacy of using ICI in the treatment of PML compared to other treatment modalities are needed.

## Supplementary Information

Below is the link to the electronic supplementary material.

Supplementary file1 (DOCX 67 KB)

## Data Availability

The data is available from the corresponding author upon reasonable request.
