# Supplementary material for: Immune checkpoints inhibitors in the treatment of progressive multifocal leukoencephalopathy: A systematic review and meta-analysis
Source: Neurol Sci. 2026 Nov 4;47(11):909. doi: 10.1007/s10072-026-09498-z (PMC13736357; doi:10.1007/s10072-026-09498-z)
Supplement: Supplementary file 1 [file 10072_2026_9498_MOESM1_ESM.docx]

**Search strategy:**

**PubMed**

( "Progressive Multifocal Leukoencephalopathy"[Mesh] OR "progressive multifocal leukoencephalopathy"[tiab] OR PML[tiab] OR "JC virus"[tiab] OR "JC polyomavirus"[tiab] OR "John Cunningham virus"[tiab] OR "JCV"[tiab] ) AND ( "Immune Checkpoint Inhibitors"[Mesh] OR "immune checkpoint inhibitor*"[tiab] OR "checkpoint inhibitor*"[tiab] OR "immune checkpoint blockade"[tiab] OR "checkpoint blockade"[tiab] OR "immune checkpoint therapy"[tiab] OR "immune checkpoint treatment"[tiab] OR "checkpoint immunotherapy"[tiab] OR "PD-1 inhibitor*"[tiab] OR "PD1 inhibitor*"[tiab] OR "PD-L1 inhibitor*"[tiab] OR "PDL1 inhibitor*"[tiab] OR "CTLA-4 inhibitor*"[tiab] OR "CTLA4 inhibitor*"[tiab] OR pembrolizumab[tiab] OR nivolumab[tiab] OR cemiplimab[tiab] OR dostarlimab[tiab] OR atezolizumab[tiab] OR durvalumab[tiab] OR avelumab[tiab] OR ipilimumab[tiab] OR tremelimumab[tiab] )

**CENTRAL:**

( "progressive multifocal leukoencephalopathy" OR PML OR "JC virus" OR "JC polyomavirus" OR "John Cunningham virus" OR JCV ) AND ( "immune checkpoint inhibitor*" OR "checkpoint inhibitor*" OR "immune checkpoint blockade" OR "checkpoint blockade" OR "immune checkpoint therapy" OR "immune checkpoint treatment" OR "checkpoint immunotherapy" OR "PD-1 inhibitor*" OR "PD1 inhibitor*" OR "PD-L1 inhibitor*" OR "PDL1 inhibitor*" OR "CTLA-4 inhibitor*" OR "CTLA4 inhibitor*" OR pembrolizumab OR nivolumab OR cemiplimab OR dostarlimab OR atezolizumab OR durvalumab OR avelumab OR ipilimumab OR tremelimumab )

**Scopus:**

TITLE-ABS-KEY( "progressive multifocal leukoencephalopathy" OR PML OR "JC virus" OR "JC polyomavirus" OR "John Cunningham virus" OR JCV ) AND TITLE-ABS-KEY( "immune checkpoint inhibitor*" OR "checkpoint inhibitor*" OR "immune checkpoint blockade" OR "checkpoint blockade" OR "immune checkpoint therapy" OR "immune checkpoint treatment" OR "checkpoint immunotherapy" OR "PD-1 inhibitor*" OR "PD1 inhibitor*" OR "PD-L1 inhibitor*" OR "PDL1 inhibitor*" OR "CTLA-4 inhibitor*" OR "CTLA4 inhibitor*" OR pembrolizumab OR nivolumab OR cemiplimab OR dostarlimab OR atezolizumab OR durvalumab OR avelumab OR ipilimumab OR tremelimumab )

**Embase:**

( 'progressive multifocal leukoencephalopathy'/exp OR 'progressive multifocal leukoencephalopathy':ti,ab OR pml:ti,ab OR 'jc virus':ti,ab OR 'jc polyomavirus':ti,ab OR 'john cunningham virus':ti,ab OR jcv:ti,ab ) AND ( 'immune checkpoint inhibitor'/exp OR 'immune checkpoint inhibitor*':ti,ab OR 'checkpoint inhibitor*':ti,ab OR 'immune checkpoint blockade':ti,ab OR 'checkpoint blockade':ti,ab OR 'immune checkpoint therapy':ti,ab OR 'checkpoint immunotherapy':ti,ab OR 'pd-1 inhibitor*':ti,ab OR 'pd1 inhibitor*':ti,ab OR 'pd-l1 inhibitor*':ti,ab OR 'pdl1 inhibitor*':ti,ab OR 'ctla-4 inhibitor*':ti,ab OR 'ctla4 inhibitor*':ti,ab OR pembrolizumab:ti,ab OR nivolumab:ti,ab OR cemiplimab:ti,ab OR dostarlimab:ti,ab OR atezolizumab:ti,ab OR durvalumab:ti,ab OR avelumab:ti,ab OR ipilimumab:ti,ab OR tremelimumab:ti,ab )

**Supplementary references**(1-52)

1.	Lambert N, Dauby S, Dive D, Sadzot B, Maquet P. Atezolizumab Treatment for Progressive Multifocal Leukoencephalopathy. Emerging infectious diseases. 2022;28(1):253-6.

2.	Kapadia RK, Ney D. Stabilization of Progressive Multifocal Leukoencephalopathy After Pembrolizumab Treatment. The Neurohospitalist. 2020;10(3):238-9.

3.	Chiuchiarelli M, Micheli G, Segala FV, Giuliano G, Del Giacomo P, Dusina A, et al. Prolonged survival in HIV-associated Progressive Multifocal Leukoencephalopathy treated with Pembrolizumab: a case series on treatment and long-term follow-up. Journal of neurovirology. 2025;31(2):109-15.

4.	Johnson T, Zoghlin L. Autoimmune Hematologic Complication of Immune Checkpoint Inhibition Treatment in a Case of Progressive Multifocal Leukoencephalopathy (P8-8.002). 2025;104(7_Supplement_1):4292.

5.	Mohamed A, Wallace G, Shi H, Henson J, Rutkowski E, Moore-Hill D. PML on Belimumab Therapy and Treatment with Pembrolizumab. 2024;103(7_Supplement_1):S1-S.

6.	Öztürk G, Tekeli E, Erdoğan S, Peker E, Yücesan C. Progressive multifocal leukoencephalopathy in sarcoidosis successfully treated with pembrolizumab. Journal of neurovirology. 2024;30(4):441-4.

7.	Ercan MB, Kocer B, Altiparmak T, Arslan I. Responsiveness to pembrolizumab in severe early-onset natalizumab associated PML-IRIS in patient with relapsing-remitting multiple sclerosis. 2024;30(6):760-2.

8.	Jeantin L, Shor N, Coustans M, Roos-Weil D, Quintin-Roué I, Bellanger A, et al. Progressive multifocal leukoencephalopathy in patients with chronic liver disease successfully treated with pembrolizumab. Journal of neurology. 2024;271(4):2119-24.

9.	Rothenberg A, Robers M, Hoskin J. Progressive Multifocal Leukoencephalopathy (PML) in an Immunocompetent Host with Excellent Clinical Response to Pembrolizumab Therapy: A Case Report. 2024;103(7_Supplement_1):S144-S5.

10.	Hamlyn E, Patel K, Kamvumbi T, Hamzah L, Monk E, Hamlyn E, et al. Use of Pembrolizumab for treatment of HIV related Progressive Multifocal Leucoencephalopathy - A pan-London case series. HIV Medicine. 2024;25:58.

11.	Jeantin L, Shor N, Pallix-Guyot M, Roos-Weil D, Bellanger A, Le Garff-Tavernier M, et al. Halting progressive multifocal leukoencephalopathy with pembrolizumab: the case of a patient with multiple sclerosis under fingolimod. Journal of neurology. 2024;271(2):729-32.

12.	Varmpompiti K, Westwood AJ, Ben-Joseph A, Sibtain N, Ibrahim MAA, Stanton B, et al. Progressive multifocal leukoencephalopathy secondary to idiopathic CD4 lymphocytopenia treated with pembrolizumab. Journal of neuroimmunology. 2023;385:578248.

13.	Fan S, Liu M, Bai L, Chen S, Hou B, Lin N, et al. Pembrolizumab for the treatment of progressive multifocal leukoencephalopathy in China. Journal of neurovirology. 2023;29(6):692-8.

14.	Sheng G, Butte M, Garcia-Lloret M, Anderson L. Progressive multifocal leukoencephalopathy (PML) treated with pembrolizumab in a patient with mosaic STAT3 gain-of-function mutation. Clinical immunology (Orlando, Fla). 2023;250:109453.

15.	Boumaza X, Bonneau B, Roos-Weil D, Pinnetti C, Rauer S, Nitsch L, et al. Progressive Multifocal Leukoencephalopathy Treated by Immune Checkpoint Inhibitors. Annals of neurology. 2023;93(2):257-70.

16.	Barritt AW, Das E, Morley N, Seymour M, Saha R, Vera J, et al. Management approach including pembrolizumab for fingolimod-associated progressive multifocal leukoencephalopathy in a patient with relapsing-remitting multiple sclerosis. 2023;29(2):301-6.

17.	Hanna S, Thomas A. NCMP-22. TREATING PROGRESSIVE MULTIFOCAL LEUKOENCEPHALOPATHY (PML) WITH PEMBROLIZUMAB: A SINGLE INSTITUTION EXPERIENCE. Neuro-Oncology. 2022;24(Supplement_7):vii195-vii6.

18.	Varmpompiti K, Wannop K, Mantoan L. Three-dose Pembrolizumab Treatment for Progressive Multifocal Leukoencephalopathy: a case report. European Journal of Neurology. 2022;29:752.

19.	Robinson R, Ramsay S, Burns P, Flynn P, Herron B, Poots J, et al. A CASE OF NATALIZUMAB-RELATED PROGRESSIVE MULTIFOCAL LEUKOENCEPHALOPATHY TREATED WITH PEMBROLIZUMAB. Journal of Neurology, Neurosurgery and Psychiatry. 2022;93(6):133.

20.	Lambert N, El Moussaoui M, Ritacco C, Moïse M, Paulus A, Delvenne P, et al. Killing Two Birds With One Stone: Effective Control of Both Non-Small Cell Lung Cancer and Progressive Multifocal Leukoencephalopathy With Atezolizumab, A Case Report. 2022;Volume 13 - 2022.

21.	Pinnetti C, Cimini E, Vergori A, Mazzotta V, Grassi G, Mondi A, et al. Use of Pembrolizumab for Treatment of Progressive Multifocal Leukoencephalopathy in People Living with HIV. Viruses. 2022;14(5).

22.	Sim BZ, Smith LK, Yarwood T, Smith S. Successful treatment of HIV-associated progressive multifocal leukoencephalopathy with pembrolizumab. AIDS (London, England). 2022;36(3):483-5.

23.	Volk T, Warnatz K, Marks R, Urbach H, Schluh G, Strohmeier V, et al. Pembrolizumab for treatment of progressive multifocal leukoencephalopathy in primary immunodeficiency and/or hematologic malignancy: a case series of five patients. Journal of neurology. 2022;269(2):973-81.

24.	Darcy S, Alexander M, McCarthy A, O'Dowd S. Pembrolizumab treatment of inflammatory progressive multifocal leukoencephalopathy: a report of two cases. Journal of neurovirology. 2022;28(1):145-50.

25.	Locati LD, Serafini MS, Carenzo A, Canevari S, Perrone F, Orlandi E, et al. Complete Response to Nivolumab in Recurrent/Metastatic HPV-Positive Head and Neck Squamous Cell Carcinoma Patient After Progressive Multifocal Leukoencephalopathy: A Case Report. 2022;Volume 11 - 2021.

26.	Chatterjee T, Roy M, Lin RC, Almoujahed MO, Ahmad S. Pembrolizumab for the treatment of Progressive Multifocal Leukoencephalopathy (PML) in a patient with AIDS: A case report and literature review. IDCases. 2022;28:e01514.

27.	Mackenzie S, Shafat M, Roddy H, Hyare H, Neill L, Marzolini MA, et al. Pembrolizumab for the treatment of progressive multifocal leukoencephalopathy following anti‐CD19 CAR‐T therapy: a case report. 2021;2(4):848-53.

28.	Lan TY, Chen YS, Cheng CF, Huang ST, Shen CY, Hsu PN. Progressive multifocal leukoencephalopathy in systemic lupus erythematosus managed with pembrolizumab: A case report with literature review. Lupus. 2021;30(11):1849-55.

29.	Coltart H, Mulka L, El Bouzidi K, Hamlyn E. Pembrolizumab treatment for progressive multifocal leukoencephalopathy immune reconstitution inflammatory syndrome. HIV Medicine. 2021;22(SUPPL 3):305.

30.	Segala FV, Lombardi F, Gaudino S, Agrati C, Cingolani A. Prolonged survival despite HIV reactivation during treatment of progressive multifocal leukoencephalopathy with Pembrolizumab. HIV Medicine. 2021;22(SUPPL 3):306-7.

31.	Fischbach L, Bauer T, Rüber T, Grobe-Einsler M, Sitter A, Radbruch A, et al. Treatment of progressive multifocal Leukoencephalopathy associated with idiopathic lymphocytopenia with Nivolumab. Journal of the Neurological Sciences. 2021;427.

32.	Beudel M, Rövekamp F, van de Beek D, Brouwer M. Single-Dose Pembrolizumab Treatment for Progressive Multifocal Leukoencephalopathy. 2021;8(4):e1021.

33.	Roos-Weil D, Weiss N, Guihot A, Uzunov M, Bellanger A, Eymard B, et al. Immune checkpoint inhibitors for progressive multifocal leukoencephalopathy: a new gold standard? Journal of neurology. 2021;268(7):2458-65.

34.	Haroche J, Cohen Aubart F, Mokhtari K, Seilhean D, Benameur N, Peyre M, et al. Immune Reconstitution Syndrome After Nivolumab for Progressive Multifocal Leukoencephalopathy. 2021;11(3):e376-e8.

35.	Patel A, Sul J, Gordon ML, Steinklein J, Sanguinetti S, Pramanik B, et al. Progressive Multifocal Leukoencephalopathy in a Patient With Progressive Multiple Sclerosis Treated With Ocrelizumab Monotherapy. JAMA neurology. 2021;78(6):736-40.

36.	Stögbauer J, Schulz-Schaeffer W, Mühl-Benninghaus R, Lochner P. Clinical and magnetic resonance imaging monitoring in progressive multifocal leukoencephalopathy treated with pembrolizumab: a case report. Neurological Sciences. 2021;42(1):357-9.

37.	Togher Z, Fullam S, Gunko A, McGuigan C. Clinical Improvement of Progressive Multifocal Leukoencephalopathy Following Pembrolizumab Infusion: a Case Report. SN Comprehensive Clinical Medicine. 2021;3(1):320-2.

38.	Möhn N, Wattjes MP, Adams O, Nay S, Tkachenko D, Salge F, et al. PD-1-inhibitor pembrolizumab for treatment of progressive multifocal leukoencephalopathy. Therapeutic advances in neurological disorders. 2021;14:1756286421993684.

39.	Holmes A, Wellings T, Walsh O, Rowlings P. Progressive multifocal leukoencephalopathy associated with a lymphoproliferative disorder treated with pembrolizumab. Journal of neurovirology. 2020;26(6):961-3.

40.	Dufour I, Duprez T, Wertz M, Saussoy P, Ackermans N, El Sankari S, et al. Improvement in progressive multifocal leukoencephalopathy after pembrolizumab-induced immune reconstruction inflammatory syndrome in a patient with follicular lymphoma. EJHaem. 2020;1(2):585-8.

41.	Mozo Ruiz M, Rosado Barrasa N, Tena Gómez D, Torralba González de Suso M. Pembrolizumab treatment for progressive multifocal leukoencephalopathy in a patient with Human Immunodeficiency Virus infection. Enfermedades Infecciosas y Microbiologia Clinica. 2020;38(8):396-7.

42.	Grassl N, Bunse L, Beutel T, Klockziem M, Gass A, Platten M, et al. Nivolumab for treatment of progressive multifocal leukoencephalopathy in Sézary syndrome. 2020;27(11):2373-4.

43.	Goereci Y, Schweitzer F, Wellstein A, Silling S, Borchmann S, von Tresckow B, et al. Clearance of JC polyomavirus from cerebrospinal fluid following treatment with interleukin-2 and pembrolizumab in an individual with progressive multifocal leukoencephalopathy and no underlying immune deficiency syndrome. 2020;27(11):2375-7.

44.	Chu L, Clift F. Pembrolizumab for progressive multifocal leukoencephalopathy in a patient with myelodysplasia and graft-versus-host disease. European Journal of Neurology. 2020;27:1273-4.

45.	Pawlitzki M, Schneider-Hohendorf T, Rolfes L, Meuth SG, Wiendl H, Schwab N, et al. Ineffective treatment of PML with pembrolizumab. 2019;6(6):e627.

46.	Küpper C, Heinrich J, Kamm K, Bücklein V, Rothenfusser S, Straube A. Pembrolizumab for progressive multifocal leukoencephalopathy due to primary immunodeficiency. Neurology(R) neuroimmunology & neuroinflammation. 2019;6(6):e628.

47.	Walter O, Treiner E, Bonneville F, Mengelle C, Vergez F, Lerebours F, et al. Treatment of Progressive Multifocal Leukoencephalopathy with Nivolumab. 2019;380(17):1674-6.

48.	Rauer S, Marks R, Urbach H, Warnatz K, Nath A, Holland S, et al. Treatment of Progressive Multifocal Leukoencephalopathy with Pembrolizumab. 2019;380(17):1676-7.

49.	Cortese I, Muranski P, Enose-Akahata Y, Ha SK, Smith B, Monaco M, et al. Pembrolizumab Treatment for Progressive Multifocal Leukoencephalopathy. The New England journal of medicine. 2019;380(17):1597-605.

50.	Hoang E, Bartlett NL, Goyal MS, Schmidt RE, Clifford DB. Progressive multifocal leukoencephalopathy treated with nivolumab. Journal of neurovirology. 2019;25(2):284-7.

51.	Medrano C, Vergez F, Mengelle C, Faguer S, Kamar N, Del Bello A. Effectiveness of Immune Checkpoint Inhibitors in Transplant Recipients with Progressive Multifocal Leukoencephalopathy. Emerging infectious diseases. 2019;25(11):2145-7.

52.	Ørum M, Lund Laursen A, Troldborg A. Progressive multifocal leukoencephalopathy in systemic lupus erythematosus treated with pembrolizumab. BMJ case reports. 2024;17(9).
